# Worry and mental health in the Covid-19 pandemic: vulnerability factors in the general Norwegian population

**DOI:** 10.1186/s12889-021-10927-1

**Published:** 2021-05-17

**Authors:** Ines Blix, Marianne Skogbrott Birkeland, Siri Thoresen

**Affiliations:** Norwegian Centre for Violence and Traumatic Stress Studies (NKVTS), Pb.181 Nydalen, 0409 Oslo, Norway

**Keywords:** COVID-19, Pandemic, Mental health, Worry

## Abstract

**Background:**

There is an urgent need for knowledge about the mental health consequences of the ongoing pandemic. The aim of this study was to identify vulnerability factors for psychological distress and reduced life satisfaction in the general population. Furthermore, we aimed to assess the role of COVID-related worries for psychological distress and life satisfaction.

**Methods:**

A presumed representative sample for the Norwegian population (*n* = 1041, response rate = 39.9%) responded to a web-survey in May 2020. The participants were asked about potential vulnerability factors including increased risk for severe illness from COVID-19 (underlying illness, older age), socioeconomic disadvantage (living alone, unemployment, economic problems), and pre-existing mental health vulnerability (recent exposure to violence, previous mental health challenges). Additional measures included COVID-related worry, psychological distress, and life satisfaction.

**Results:**

More than one out of four reported current psychological distress over the threshold for clinically significant symptoms. Socioeconomic disadvantages, including living alone and pre-existing economic challenges, and pre-existing mental health vulnerabilities, including recent exposure to violence and previous mental health problems, were associated with a higher level of psychological distress and a lower level of life satisfaction. A higher level of COVID-related worry was significantly associated with a higher level of psychological distress, and a lower level of life satisfaction, even when adjusting for all the vulnerability factors.

**Conclusion:**

This study identified several vulnerability factors for mental health problems in the pandemic. Individuals recently exposed to violence and individuals with pre-existing mental health problems are at particular risk. Worrying about the consequences of the pandemic contributes negatively to current mental health. However, worry cannot explain the excess distress in vulnerable groups. Future research should focus on how COVID-related strains contribute to mental health problems for vulnerable groups.

**Supplementary Information:**

The online version contains supplementary material available at 10.1186/s12889-021-10927-1.

## Background

Emerging evidence shows that the COVID-19 pandemic not only poses a threat to physical health but also to mental health in the community. The pandemic has profound effects on our daily life, and will for many cause major stressors, including the fear of the disease itself, social distancing, and isolation due to the mitigation strategies implemented by the authorities, as well as economic consequences of the mitigation strategies. These stressors may cause worries and influence mental health. A recent review showed high prevalence rates for stress, anxiety, and depression in the general population, across studies more than a third of the respondents scored above the threshold for anxiety and depression [1]. One study from Norway reported two to threefold increases in anxiety and depression symptoms, compared to pre-pandemic levels [2]. Although most individuals show resilience and do not experience substantial psychological distress due to the pandemic, some groups in our society may carry a heavier psychosocial burden. As pointed out by scholars in the field, there is an urgent need for research that focuses on the mental health consequences of the ongoing pandemic for the general population, and particularly for vulnerable groups [3].

The stressors during the pandemic are not evenly distributed in the population. Rather, some groups are likely to experience more stressors that can lead to psychological distress. For example, a study conducted in Spain during the lockdown in March 2020, showed that previous diagnoses of mental health problems or neurological disorders, having symptoms associated with the virus or having a close relative infected, and female sex were associated with higher levels of anxiety, depression, and posttraumatic stress reactions [4]. A study from Ireland reported that lost income, younger age, and female sex were significant predictors for anxiety or depression during the lockdown [5]. In a recent review, Xiong et al. [6] reported that among the risk factors associated with mental distress during the COVID-19 pandemic were female sex, younger age (≤40 years), and chronic/psychiatric illnesses. Despite the high variability between countries in the burden of the pandemic and the countermeasures, the research so far seems to underscore similarities in vulnerability factors for mental health problems, at least when it comes to young age and female sex. We argue that the pandemic can involve particularly strong stressors for people with increased vulnerability for serious illness due to COVID-19, people with socioeconomic disadvantages, and with pre-existing mental health problems. Further research is necessary to identify more specific vulnerability factors and their potential variation across time and place.

Research on vulnerability factors provides important knowledge about the groups that carry a higher risk for psychological distress in the ongoing pandemic. However, it is not clear to what extent these vulnerability factors are specific for the pandemic, or in what way the pandemic constitutes an additional burden for vulnerable groups. In this study, we take this research one-step further by examining the relative significance of several vulnerability factors, as well as the significance of worry for mental health and life satisfaction during the pandemic. Several studies have shown that COVID-related worry is associated with psychological distress in the pandemic [7–9]. Worry refers to problem-focused thoughts about the future, and these types of thoughts can motivate us to search for goals or solutions [10] and are initially an adaptive response to a threatening situation like the current pandemic. However, when worry becomes excessive or difficult to control, these thoughts can be experienced as negative, and lead to psychological distress [11].

COVID-related worry and psychological distress in the population are likely to vary across different places and different phases of the pandemic. Indeed, one study reported significant regional differences across the US, with a higher level of fear and worry in highly affected areas [12]. Hence, there is a need to specify factors associated with psychological distress in different places and different phases of the pandemic to prioritise and target efforts to prevent distress. The COVID-19 pandemic can be long lasting, and there is a need to prevent long-lasting mental health problems. The main aim of the present study was to identify vulnerability factors associated with higher levels of psychological distress and lower life satisfaction in the general population. More specifically, we wanted to examine the role of increased risk for severe illness from COVID-19 (due to underlying illness or old age), socioeconomic disadvantage (due to living alone, unemployment, or economic problems), and pre-existing mental health vulnerability (due to exposure to violence or previous mental health challenges). Furthermore, we wanted to assess the contribution of COVID-related worry to psychological distress and life satisfaction.

The data in the current study were collected in May 2020. At the time, the COVID-19 situation was described as under control in the Norwegian society, and the government had recently started easing the countermeasures. Schools were gradually opening for more than the four youngest cohorts from May 11th, although most schools did not open for full-day activity until the beginning of June. Most leisure activities were still closed (gyms, cinemas, museums, theatres), however many institutions aimed to reopen or partly reopen during the coming month. The government upheld the rule of physical distance to other people and the advice against non-essential public transport. Employees were instructed to work from home if possible but allowed to attend the office if necessary pending COVID-19 adaptations at the workplace. Thus, our study may reflect a snapshot of the population in a time characterized by ‘opening up’ the society.

## Methods

### Participants and procedure

The web-survey was performed by the data collection agency Kantar/Gallup Norway in their panel consisting of approximately 46,000 participants. The panel was constructed to represent the Norwegian general population in miniature. Recruitment to the panel was done by probability sampling, not self-recruitment. The panel is considered representative of the Norwegian ‘internet population’ (everyone who has access to the internet), which constitutes about 97% of the total Norwegian population. Sampling and weighting were performed based on official statics from Statistics, Norway. Sociodemographic information on panel members is updated each year. Panel members received points for their participation according to the number of minutes estimated to complete the questions. In the current study, estimated to 20 min completion time, participants were rewarded 20 points (equals 20 Norwegian Kroner, 1.9 Euro, or 2.2 USD). The data collection was performed within one week (19–26 May 2020).

In the present web survey, Kantar/Gallup approached a total of 2612 individuals stratified on sex, age, education, and area of residence. In total, 39.9% (*N* = 1041) completed the survey, 55.8 (*N* = 1457) did not respond, 2.7% (*N* = 71) started but did not complete, 1.6% (*N* = 41) clicked on the link to participate but did not confirm agreement to the terms of the study, and 0.1% (*N* = 2) withdrew from the study. Our study participants did not differ from non-responders in sex or education (Table 1), but the sample was highly skewed toward older age, with a mean age of 54.1 in responders and 43.3 in non-responders. Subgroup analyses of various age groups showed a steady trend from a poor participation rate of 17.0% in the youngest age group (age 18–29) increasing to 57.1% in the oldest (60 years and above). According to Kantar (personal communication, 2020), the problem of recruiting young adults is a general survey trend and not specific to the current study. However, caution should be taken when interpreting results for the youngest age group.

**Table 1:** Sample Characteristics for Responders and Non-Responders.

Characteristics	Respondents % (N) / mean (SD)	Non-respondents % (N) / mean (SD)	X2/t-test *p* value
Sex: Female	49.0% (510)	50.5% (794)	0.438
Age (mean)	54.1 (15.9)	43.3 (17.2)	< 0.001
Age groups			
18–29 (*n* = 524)	17.0% (89)	83.0% (435)	
30–44 (*n* = 664)	31.7% (211)	68.3% (454)	
45–59 (*n* = 659)	46.3% (305)	53.7% (354)	
60 and above (*n* = 764)	57.1% (436)	42.9% (328)	
Living alone	22.3% (229)	19.4% (294)	0.075

The Regional Committee for Medical and Health Research Ethics approved the study (Registration number 133226/2020). The questionnaire was briefly piloted before the data collection. The survey included an open box for comments and feedback on the study. The vast majority of comments were positive or even grateful, although several individuals remarked that some work-related questions were not suitable for those who had retired.

### Measures

#### Increased vulnerability for serious illness due to COVID-19 (age and underlying disease)

At-risk health condition was measured with a single question (yes/no): ‘Do you have a chronic disease or a health problem with an increased risk of serious disease from covid-19 (e.g. cancer, heart condition, diabetes)? Age was recorded by the data collection agency, age 65 and higher has been defined as a risk factor, and accordingly age was dichotomized into ‘High’ (>/=65) or to ‘Not high’ (< 65).

#### Socioeconomic disadvantages (living alone, pre-existing economic challenges, COVID-related unemployment)

The participants were asked about the number of residents living in their household, if the answer was one, this variable was coded as ‘living alone’. Perceived pre-existing economic problems were measures by asking the participants to evaluate their relative economic situation (before the pandemic) was ‘above average’, ‘average’, or ‘below average’. We dichotomized this variable into ‘below average’ and ‘average or above’. The participants were also asked if they had lost their job or being temporarily laid off due to the pandemic, if yes on any of these, this was coded as “COVID-related unemployment”.

#### Pre-existing mental health vulnerability (previous/pre-existing mental health challenges, recent exposure to violence)

Pre-existing mental health vulnerability included measures of previous mental health challenges and recent exposure to violence. To measure previous mental health challenges that the participants were asked whether or not they had previously received treatment for mental health problems. To measure exposure to violence the last month, the participants were asked if they had experienced that someone had: 1) repeatedly ridiculed you, put you down, ignored you, or told you that you were no good 2) slapped, pinched, pulled, or shook you violently, 3) hit you with a fist or a hard object, kicked, strangulated, beaten up, threatened with a weapon, or physically attacked in other ways, 4) exposed you to any form of sexual assault or violation.

#### COVID-related worry

The participants were asked to indicate their level of worry on a scale from 1 (not worried) to 7 (very worried) for 12 questions about COVID-related worries. These questions were adapted from the COSMO study [13]. In this study, we wanted to capture an underlying tendency to worry about COVID-19-related issues. Therefore, we conducted a confirmatory factor analysis (CFA) of our 12 proposed worries related to COVID-19 (see Table 2).

**Table 2:** COVID-related worry Mean (SD), factor loadings for 12 and 6 items.

Worries (1-don’t worry at all- 7-worry a lot)	Mean (SD)	Factor loadings 12-item one- factor model	Factor loadings 6-item one- factor model
*Losing someone I love	3.95 (1.63)	.70	.74
*Becoming seriously ill from the virus	3.29 (1.55)	.66	.68
*Infecting others	3.71 (1.67)	.62	.65
Not being able to get the medicines or treatment that I need	2.90 (1.64)	.62	
*Health system being overloaded	3.70 (1.55)	.74	.70
Economic recession in Norway	4.60 (1.47)	.52	
Become unemployed	2.33 (1.70)	.37	
Not be able to carry out plans that are important to me	3.54 (1.65)	.53	
*Not be able to visit people who depend on me	4.07 (1.71)	.66	.62
The society will become more egoistic	3.79 (1.66)	.52	
The welfare society will collapse?	3.54 (1.65)	.56	
*A new outbreak of COVID-19	4.30 (1.52)	.77	.79
Model fit			
χ^2^		683.51	59.38
df		54	9
CFI		0.85	0.98
RMSEA		0.11	0.07

* The six items that comprised the one-factor model. These six COVID-related worries were included in a mean total score, and used in the further analyses

The one-factor CFA with all 12 items showed poor model fit, and that several of the proposed items loaded poorly (see Table 2). We excluded items one by one until the model fit indices showed acceptable fit and all factor loadings were appropriate. We considered values of CFI above .95 and RMSEA above .08 to indicate acceptable model fit [14, 15]. Factor loadings above .70 were considered excellent .63 very good, .55 good, and .45 fair [16]. When a one-factor model showed acceptable fit, six items remained. We computed a total mean score of these six COVID-related worries and used this in the further analyses.

The CFA for COVID-related worries showed an acceptable fit and all factor loadings were appropriate when six COVID-related worries were included in the one-factor model. Means, standard deviations, model fit, and factor loadings of the 12-item one-factor model, and the final 6-item one-factor model can be seen in Table 2. As can be seen in Table 2, the highest mean level of worry was about a new outbreak of COVID-19, not be able to visit people who depend on me, and the economic recession in Norway.

*Psychological distress the last two weeks* was measured by an abbreviated 5-item version of the Hopkins Symptom Checklist-25 (HSCL) [17]: Feeling hopeless about the future; feeling blue; worrying too much about things; feeling fearful; feeling tense or worked up. Participants responded on a scale from 0 (not bothered) to 3 (bothered a great deal). This abbreviated version of the HSCL has shown good psychometric properties and has previously been found to correlate highly (r = 0.92) with the HSCL-25 in a general population sample [18]. Based on previous research we used a cut-off value of > 2, which has achieved the best combination of specificity, sensitivity, and predictive values for clinically significant symptoms [19]. Cronbach’s Alpha for the 5-item HSCL was 0.91 in the present study.

*Life satisfaction* was measured by a single question from the European Social Survey (https://www.europeansocialsurvey.org/): ‘All things considered, how satisfied are you with your life as a whole nowadays?’ Participants responded on a scale from 0 (Extremely dissatisfied) to 10 (Extremely satisfied).

### Statistical analyses

Univariate and multivariate linear regression analyses were performed with psychological distress and life satisfaction as the dependent variables. Univariate linear regression analyses were performed with pre-existing health problems, age older than 65, living alone, pre-existing economic challenges, becoming unemployed during the pandemic, exposure to violence the last month, previous mental health challenges, and female sex entered separately as independent variables and psychological distress and life satisfaction as dependent variables. The same independent variables were included simultaneously in two multivariate linear regression analyses, with psychological distress and life satisfaction as dependent variables. COVID-related worry was added as an independent variable together with the above-mentioned variables. Statistical analyses were conducted with SPSS version 26 for Windows (SPSS, Inc.) and Mplus 8.3.

## Results

Of the 1041 participants, 49.0% (*n* = 510) were females. The age range was between 18 and 89 years old, with a mean of 54.1 (SD = 15.9). A percentage of 35.9% (*n* = 374) had college/university education. 25.7% (268 participants) reported current psychological distress over the threshold for clinically significant symptoms.

Table 3 shows the mean number and percentage of the participants that reported each of the potential vulnerability factors. Old age (> 65 years), underlying illness, living alone, and pre-existing mental health problems were the most frequent potential vulnerability factors. For each of the vulnerability factors, and the sample in total, mean scores for psychological distress, COVID-related worry, and life satisfaction are reported. Individuals who had recently been exposed to violence had pre-existing mental health problems, or pre-existing economic challenges reported particularly high levels of psychological distress.

**Table 3:** Vulnerability factors, n (%), mean (SD) for psychological distress, COVID-related worry and life satisfaction.

Vulnerability factors		N (%)	COVID-related worry mean (SD)	Psychological distress mean (SD)	Life satisfaction mean (SD)
**At risk for severe illness from COVID-19**					
Underlying illness	Yes	255 (24.5)	4.07 (1.31)	1.62 (.66)	7.69 (1.80)
	No	766 (73.6)	3.75 (1.16)	1.60 (.68)	7.90 (1.80)
Old age (> 65 years)	Yes	307 (29.5)	3.67 (1.29)	1.42 (.45)	8.29 (1.55)
	No	732 (70.3)	3.90 (1.17)	1.69 (.73)	7.66 (1.86)
**Socioeconomic disadvantage**					
Living alone	Yes	231 (22.2)	3.90 (1.31)	1.76 (.74)	7.42 (1.98)
	No	808 (77.6)	3.81 (1.18)	1.57 (.65)	7.97 (1.72)
Pre-existing economic challenges	Yes	129 (12.5)	4.17 (1.20)	2.05 (.84)	6.61 (1.98)
	No	895 (86.0)	3.78 (1.20)	1.54 (.62)	8.03 (1.67)
COVID-related unemployed	Yes	109 (10.5)	3.80 (1.27)	1.80 (.82)	7.72 (2.11)
	No	931 (89.4)	3.84 (1.20)	1.59 (.65)	7.86 (1.76)
**Pre-existing mental health vulnerability**					
Recent violence exposure	Yes	59 (5.7)	4.45 (1.35)	2.46 (.77)	6.78 (2.41)
	No	980 (94,1)	3.78 (1.19)	1.56 (.63)	7.91 (1.73)
Pre-existing mental health problems	Yes	223 (21.4)	4.01 (1.30)	2.06 (.81)	7.14 (2.01)
	No	807 (77.5)	3.78 (1.18)	1.49 (.58)	8.04 (1.69)
**Total sample**			4.30 (1.52)	1.61 (.67)	7.84 (1.80)

Linear regression analyses showed that living alone, pre-existing economic challenges, being exposed to violence within the last month, previous mental health problems, and female sex, were significantly associated with a higher level of psychological distress. Conversely, age higher than 65 years was negatively associated with psychological distress. When mutually adjusted (model 1, Table 4), all the same factors, except COVID-related unemployment, were independently associated with a higher level of psychological distress.

**Table 4:** Unadjusted and adjusted linear regression analyses for vulnerability factors and COVID-related worries predicting psychological distress.

	Unadjusted				Model 1: adjusted				Model 2: adjusted, worry as predictor			
	B	95% CI	p	β	B	95% CI	p	β	B	95% CI	p	β
**At risk for severe illness from COVID-19**												
Underlying illness	.03	−.07,.12	.591	.02	.03	−.06, .12	.497	.03	−.03	−.11,.05	.467	−.02
Older age (> 65 years)	−.28	−.36,-.19	<.001	−.18	−.16	−.25, −.08	<.001	−.11	−.13	−.21–.05	.002	−.09
**Socioeconomic disadvantage**												
Living alone	.19	.09,.29	<.001	.12	.11	.02,.20	.018	.07	.12	.03,.20	.028	.07
Pre-existing economic challenges	.52	.40,.64	<.001	.26	.37	.26,.48	<.001	.18	.32	.22,.43	<.001	.16
COVID-related unemployed	.21	.08,.34	.002	.10	.04	−.08,.16	.641	.04	.06	−.06,.17	.318	.03
**Pre-existing mental health vulnerability**												
Recent violence exposure	.91	.74,1.08	<.001	.31	.70	.54,.87	<.001	.24	.57	.42,.73	<.001	.20
Pre-existing mental health problems	.57	.47,.66	<.001	.35	.42	.33,.51	<.001	.26	.41	.32,.49	<.001	.26
Female sex	.17	.09,.26	<.001	.13	.12	.05,.19	.001	.09	.06	−.01,.13	.113	.04
COVID-related worries									.17	.14,.20	<.001	.31
R square							.25			.33		

*Unadjusted: all predictors were entered separately. Model 1: all predictors except COVID-related worries were included simultaneously. Model 2: COVID-related worry was entered while adjusting for all the vulnerability factors

Adding COVID-related worry as a predictor in the multivariate regression analysis (model 2, Table 4) did not substantially attenuate the associations between the suggested vulnerability factors and psychological distress. All the same factors, except female sex, were still significantly associated with distress. The magnitude of the regression coefficient for recent violence exposure seemed to be lower in the adjusted model, but the confidence intervals were overlapping. A higher level of COVID-related worry was uniquely associated with a higher level of psychological distress.

Linear regression analyses showed that living alone, pre-existing economic challenges, being exposed to violence within the last month and previous mental health problems, were associated with lower levels of reported life satisfaction. Age higher than 65 years was associated with a higher level of life satisfaction. When mutually adjusted (model 1, Table 5), all the same factors and underlying illness were independently associated with a lower level of life satisfaction.

**Table 5:** Unadjusted and adjusted linear regression analyses for vulnerability factors and COVID-related worries predicting life satisfaction.

	Unadjusted				Model 1: adjusted				Model 2: adjusted, worry as predictor			
	B	95% CI	p	β	B	95% CI	p	β	B	95% CI	p	β
**At risk for severe illness from COVID-19**												
Underlying illness	−.22	−.47,.04	.095	−.05	−.27	−.52,-.03	.029	−.07	−.20	−.44,.05	.113	−.05
Older age (> 65 years)	.63	.40,.87	<.001	.16	.52	.28,.77	<.001	.13	.46	.22,.70	<.001	.13
**Socioeconomic disadvantage**												
Living alone	−.56	−.82,-.30	<.001	−.13	−.45	−.70,-.19	.001	.10	−.43	−.68,-.18	<.001	−.10
Pre-existing economic challenges	−1.41	−1.72,-.1.10	<.001	−.27	−1.18	−1.49,-.86	<.001	−.22	−1.11	−1.41,-.80	<.001	−.21
COVID-related unemployed	−.14	−.50,.22	.453	−.02	.04	−.30,-.38	.818	.01	.01	−.33,.34	.980	.01
**Pre-existing mental health vulnerability**												
Recent violence exposure	−1.13	−.1.60,-.66	<.001	−.15	−.56	−1.03,-.10	.017	−.07	−.39	−.85,.07	.096	−.05
Pre-existing mental health problems	−.90	−1.17,-.64	<.001	−.21	−.59	−.85,-.33	<.001	−.14	−.58	−.84,-.33	<.001	−.14
Female sex	−.21	−.43,.01	.057	−.06	−.10	−.31,.11	.334	−.03	−.03	−.23,.18	.816	−.01
COVID-related worries									−.24	−.32,.-15	<.001	−.16
R square						.14				.16		

*Unadjusted: all predictors were entered separately. Model 2: all predictors except COVID-related worries were included simultaneously Model 3: COVID-related worry was entered while adjusting for all the vulnerability factors

When COVID-related worry was added as a predictor in the multivariate regression analysis (model 2) the association between recent violence exposure and life satisfaction the confidence intervals were somewhat attenuated but the confidence intervals were overlapping. When adjusting for worry the association between underlying illness and life satisfaction was also lower, but with overlapping confidence intervals. Adding worry as a predictor did not considerably attenuate the associations between the other independent variables and life satisfaction. Age higher than 65 years was associated with a higher level of life satisfaction. The results showed that a higher level of COVID-related worry was uniquely associated with a lower level of life satisfaction.

We performed the same analyses, weighted on sex, age, education, and area of residence, and these showed very similar results.

## Discussion

A substantial proportion of the general Norwegian population experienced significant psychological distress in the first opening-up phase of the COVID-19 pandemic. Indeed, more than one out of four reported current psychological distress over the threshold for clinically significant symptoms. Whereas population data from 2019 show that 14% scored above the clinical threshold [20]. Similar findings have been reported in studies from Ireland [5], the UK [21], and Denmark [22]. It should be noted that at the time of the data collection, in May 2020, Norway was not hit particularly hard by the pandemic compared to other countries as indicated by a relatively low number of fatalities [23] and sufficient hospital intensive care capacity to handle the comparably modest number of covid-19 patients. Additionally, the countermeasures were not among the strictest in a European context, for example, a curfew was not implemented. Thus, the present study adds to the literature by showing that the pandemic can lead to increased psychological distress in the population, even in a situation with low infection rates and moderate countermeasures.

Some groups may carry a heavier psychosocial burden in the pandemic. In this study, we examined the role of three types of vulnerability factors: increased risk for severe illness from COVID-19 (due to underlying illness or older age), socioeconomic disadvantage (due to living alone, unemployment or economic problems), and pre-existing mental health vulnerability (due to exposure to violence or previous mental health challenges). Taken together, our findings showed that socioeconomic disadvantage and pre-existing mental health problems, recent violence exposure, but no increased risk for severe illness, was associated with a higher level of psychological distress and a lower level of life satisfaction. Results from this study thus inform the design of prevention measures in identifying target groups that may be particularly vulnerable to the stress and strains of the pandemic.

Contrary to our expectations, we found no evidence for an association between increased risk for severe illness from COVID-19, and a higher level of psychological distress. This contrasts with previous studies from Turkey conducted in April 2020 [24], from Spain conducted in March 2020 [25] and from Italy conducted in March 2020 [26], which all reported that chronic disease was associated with higher levels of psychological distress. However, it is important to note that the context and phase of the pandemic were different across these studies. Whereas our study was performed in an opening up phase, in a context of moderate countermeasures, and low levels of fatalities, the studies in Italy, Spain, and Turkey were conducted in a situation where the pandemic was rising, the intensive care capacities were challenged and there was a high level of fatalities. Likely, individuals living with increased risk for severe illness due to COVID-19 would experience a higher level of perceived threat in a situation where the pandemic is currently not under control.

Older age was not identified as a vulnerability factor for psychological distress. On the contrary, the results showed that older age was associated with a lower level of psychological distress, and a higher level of life satisfaction. Similar findings were reported by González-Sanguino an colleagues [4]. Other studies conducted during the COVID-19 pandemic have also reported that younger, but not older age is associated with a higher level of psychological distress [6]. In a diary study, from March/April 2020, Klaiber, Wen, DeLongis, and Sin [27] reported that older and younger participants experienced comparable levels of stress, but younger participants expressed more worry. It should be mentioned that the tendency for older adults to worry less than younger adults have also been found in previous studies conducted outside a pandemic context [28, 29]. Furthermore, several studies have also shown that life satisfaction increases with older age in adulthood [30]. Maybe older individuals are more able to cope with the uncertain situation that the COVID-19 pandemic represents and that more is at stake for young people and their future. Indeed, older adults are more experienced in coping with problems and it has been suggested that older adults are more capable of regulating their emotions [31].

The present results showed that socioeconomic disadvantages, including living alone and pre-existing economic challenges, were associated with a higher level of psychological distress, and a lower level of life satisfaction. This illustrates the disproportionate impact of the pandemic and the significance of where pre-existing resources. Some individuals have lower access to the safety net of economic and other resources, and for these people, the pandemic constitutes a risk for further losses and over time, and further social marginalisation. Pre-existing mental health vulnerabilities, including recent exposure to violence and previous mental health problems, were associated with a higher level of psychological distress and a lower level of life satisfaction. Previous studies have also shown that previous or pre-existing mental health problems are a vulnerability factor during the pandemic (for a review see Xiong et al. 2020), but to our knowledge, the present study was the first to investigate recent exposure to violence as a vulnerability factor. It has been discussed whether lockdown, closed schools, and social isolation may result in domestic violence. Although this study could not compare violence exposure to previous levels, our results underscore the importance of targeting this group for prevention purposes. Many of the violence-exposed individuals may already be known within health services and child protection services, and outreach services may be suitable to ensure their safety and support their coping. These present findings highlight the need to prevent discontinuation of activities or treatment for individuals with pre-existing mental health vulnerabilities. As pointed out by Galea et al. [32], efforts should be made to facilitate connection with individuals at risk for social marginalisation during the pandemic.

Although worrying initially is an adaptive response to the pandemic, the present results suggest that COVID-related worry can play an important role in psychological distress and life satisfaction in the pandemic. Even when adjusting for all the vulnerability factors, a higher level of COVID-related worry was significantly associated with a higher level of psychological distress, and a lower level of life satisfaction. This is in line with previous studies reporting a link between COVID-related worry and psychological distress [7–9]. The present study extends these findings by examining the role of worry for psychological distress in vulnerable groups.

However, the results showed that COVID-related worry can only to a small extent explain the higher levels of psychological distress and lower level of life satisfaction among vulnerable groups. The vulnerability factors were independently associated with psychological distress and lower level of life satisfaction. Individuals with these vulnerabilities may also have less access to resources such as social support, which might have an alleviating effect on the strains of the pandemic. Thus, both individuals with and without vulnerabilities can worry about the pandemic so much that it brings about psychological distress and decrease their quality of life. COVID-related worry seems to partly explain why women report more distress in the pandemic. However, the present results do not suggest that COVID-related worry can explain the excess distress in vulnerable groups. For example, our results do not indicate that it because of COVID-related worry that an individual exposed to recent violence is more distressed. Other factors, not included in our study, maybe more important, such as loss of social network, loss of treatment contacts, unavailability of child protection services, or increased threat to financial security.

The present study has some strengths and limitations. A stratified probability sample, with a 39.9% response rate adds to the strengths of the study. Although we cannot exclude a self-selection bias, we could assess the representability by comparing demographic characteristics between responders and non-responders. We found that our sample was skewed in terms of high age. However, analyses weighted for age provided similar results. It should also be mentioned that systematic response rate biases are more likely to affect levels of vulnerability factors, COVID-related worries, psychological distress, and life satisfaction than estimates of their associations. Additionally, it is important to emphasise that the present study is cross-sectional and only shows a snapshot of COVID-related worries, life satisfaction, and psychological distress at a particular time and place. In the present study, we initially asked about different types of worries related to COVID-19, and the results of the CFA identified health-related worries as a unitary factor. However, other types of worries that we did not measure are probably also important for psychological distress and life satisfaction. Different types of worries will probably affect different vulnerable groups, and the most prevalent worries will probably change with time, local context, political and economic circumstances.

## Conclusion

In conclusion, our study identified several unique vulnerability factors for psychological distress and lower life satisfaction in the pandemic. In particular, individuals recently exposed to violence, and individuals with pre-existing mental health problems were at risk. Worrying about the consequences of the pandemic can contribute negatively to current mental health. However, worry does not seem to explain the excess distress in vulnerable groups. The COVID-19 pandemic is still ongoing and is likely to continue to affect us for a long time to come. An understanding of how the pandemic develops through several phases and how vulnerabilities and worries may change throughout the pandemic will benefit people who struggle with strains and worries. Interventions should specifically target vulnerable groups to prevent long-term suffering. Future research should make an effort to identify which COVID-related strains make life so difficult for vulnerable groups in the pandemic.

## Supplementary Information

**Additional file 1.** Questionnaire- questions used in the present paper.

## Data Availability

The data are not publicly available due to them containing information that could compromise research participant privacy and consent.

## Abbreviations

CFA: Confirmatory factor analysis
COVID: corona virus disease
HSCL: Hopkins Symptom Checklist
